# Successful surgical and medical treatment of a severe, acute epidural bleed in a young dog due to steroid responsive meningitis-arteritis

**DOI:** 10.1186/s13028-021-00593-z

**Published:** 2021-07-10

**Authors:** Jessica Zilli, Agnieszka Olszewska, Daniela Farke, Martin Jürgen Schmidt

**Affiliations:** Small Animal Clinic-Neurosurgery, Neuroradiology and Clinical Neurology, Justus Liebig University-Giessen, Frankfurterstrasse 114, 35392 Giessen, Germany

**Keywords:** Canine, Cervical pain, Cytosine arabinoside, Decompression, Inflammatory disease, Immune-mediated, Tetraparesis

## Abstract

**Background:**

Steroid responsive meningitis-arteritis (SRMA) is an immune-mediated disease of the leptomeninges and its associated blood vessels, typically responsive to corticosteroids. Clinically relevant haemorrhage is a rare finding in such patients and for this reason surgical decompression of the spinal cord is normally not considered. The diagnosis of SRMA is supported by serum C-reactive protein (CRP) increase, cerebrospinal fluid (CSF) examination, including cytology (polymorphonuclear pleocytosis in the acute form), nucleated cell-, red blood cell- and protein count, as well as by evaluating CSF and serum IgA concentrations. D-dimer concentrations in serum and CSF should be elevated as well and therefore can be also evaluated as a further diagnostic tool.

**Case presentation:**

A 1.5-year-old mixed breed dog was presented with pyrexia, cervical pain and acute tetraparesis. Magnetic resonance imaging revealed an extradural mass lesion at the level of the sixth cervical vertebra, consistent with a subacute epidural haemorrhage, causing severe compression of the spinal cord. Based on the dog’s signalment, clinical history and results of the blood and CSF analyses (incl. D-dimer determination), SRMA with secondary epidural haemorrhage was suspected. Decompressive surgery was performed through a right sided partial dorsal laminectomy. Post-surgical immunosuppressive treatment was started with cytarabine and then continued with prednisolone after completion of wound healing.

**Conclusions:**

This is the first report in which medical and surgical treatment were combined in a patient with SRMA and it highlights the possibility of performing a successful surgical intervention despite the need for immunosuppressive therapy. Moreover, while SRMA diagnosis is normally based on CSF analysis and CSF and serum IgA concentrations, D-dimer concentrations in serum and CSF were also useful in this patient.

## Background

Steroid responsive meningitis-arteritis (SRMA) is an immune-mediated inflammatory disorder targeting the leptomeninges and its associated vessels [1]. An underlying mechanism is the dysregulation of the immune response leading to release of interleukin 8 and a CD11a upregulation, which act both as a chemotactic factor for polymorphonuclear leukocytes [2, 3] and a massive upregulation of immunoglobulin A (IgA), due to a Th2-mediated immune response [4]. The subsequent leakage of the blood-brain barrier and stimulation of the immune system leads to an invasion of the leptomeninges with neutrophils and to the development of a necrotising arteritis [3]. The aetiopathological mechanism which leads to the arteritis is still not completely understood. In fact, although it was hypothesised that the deposition of immune complexes in these vessels could play a role in generating vascular damage, no immune complex deposits were found in acute lesions and also the deposition of immunoglobulins was rarely observed and only in chronic cases [5].

Young, medium to large breed dogs are commonly affected. The age of onset is typically between six and 18 months with a range from 4 months to 9 years [6]. Bernese Mountain dogs, Beagles, Boxers, Nova Scotia Duck Tolling Retrievers and Weimaraners are predisposed [3]. Clinical signs include lethargy, pyrexia, severe cervical hyperesthesia and pain. A stiff gait and reluctance to walk are also common. In the chronic stage of the disease, neurological signs compatible with cervical or multifocal myelopathy can be evident [7]. The diagnosis of SRMA is supported by serum C-reactive protein (CRP) increase, cerebrospinal fluid (CSF) examination, including cytology (polymorphonuclear pleocytosis in the acute form), nucleated cell-, red blood cell- and protein count, as well as by evaluating CSF and serum IgA concentrations. Treatment consists of immunosuppressive doses of prednisolone or prednisone and is usually followed by a rapid improvement. For the acute as well as for the chronic or refractory cases, a combination of azathioprine and glucocorticoids has also proven effective [8, 9]. For relapses, the efficacy of a combination of prednisolone and cytosine arabinoside has been demonstrated as well [10]. In one study, mycophenolate mofetil has been also used for the treatment of dogs with relapses [6]. The prognosis in dogs with acute disease is good if they are adequately treated; however, relapses can occur [3]. Although structural damage to the small calibre arteries can be seen in the histopathological examination of the brain and spinal cord, clinically overt haemorrhage is rarely seen in the acute form of the disease [6, 7, 11]. Here, we report on medical and surgical treatment of spinal haemorrhage that occurred in association with SRMA.

## Case presentation

A 1.5-year-old neutered male, mix-breed dog (body weight: 20 kg) was referred to the Small Animal Hospital of the Justus-Liebig-University, Giessen due to pyrexia and a 5-day history of progressive tetraparesis, cervical hyperaesthesia, inappetence and lethargy. Treatment with metamizole, meloxicam and unrecorded antibiotics prescribed from the referring veterinarian caused only a mild improvement of clinical signs. On the initial clinical examination, the dog showed an elevated body temperature of 39.7 ℃. Neurological examination revealed non-ambulatory tetraparesis with absent proprioceptive positioning and hopping in all four limbs. Spinal reflexes were increased in the hindlimbs, whereas the flexor reflex was decreased in the left front limb. Additionally, the dog showed severe cervical pain especially by latero- and dorsiflexion of the neck. Furthermore, diffuse pain on palpation along the whole spine was observed. Neuroanatomical localisation was multifocal with spinal segments C6–Th2 mainly being suspected. A complete blood count and blood chemistry profile were performed and showed a marked leucocytosis, neutrophilia and monocytosis, moderate hyperglobulinemia and marked increase of CRP (148 mg/L; reference: < 14.9 mg/L). Radiographs of the thorax and spine as well as abdominal ultrasound were unremarkable.

**Fig. 1:**
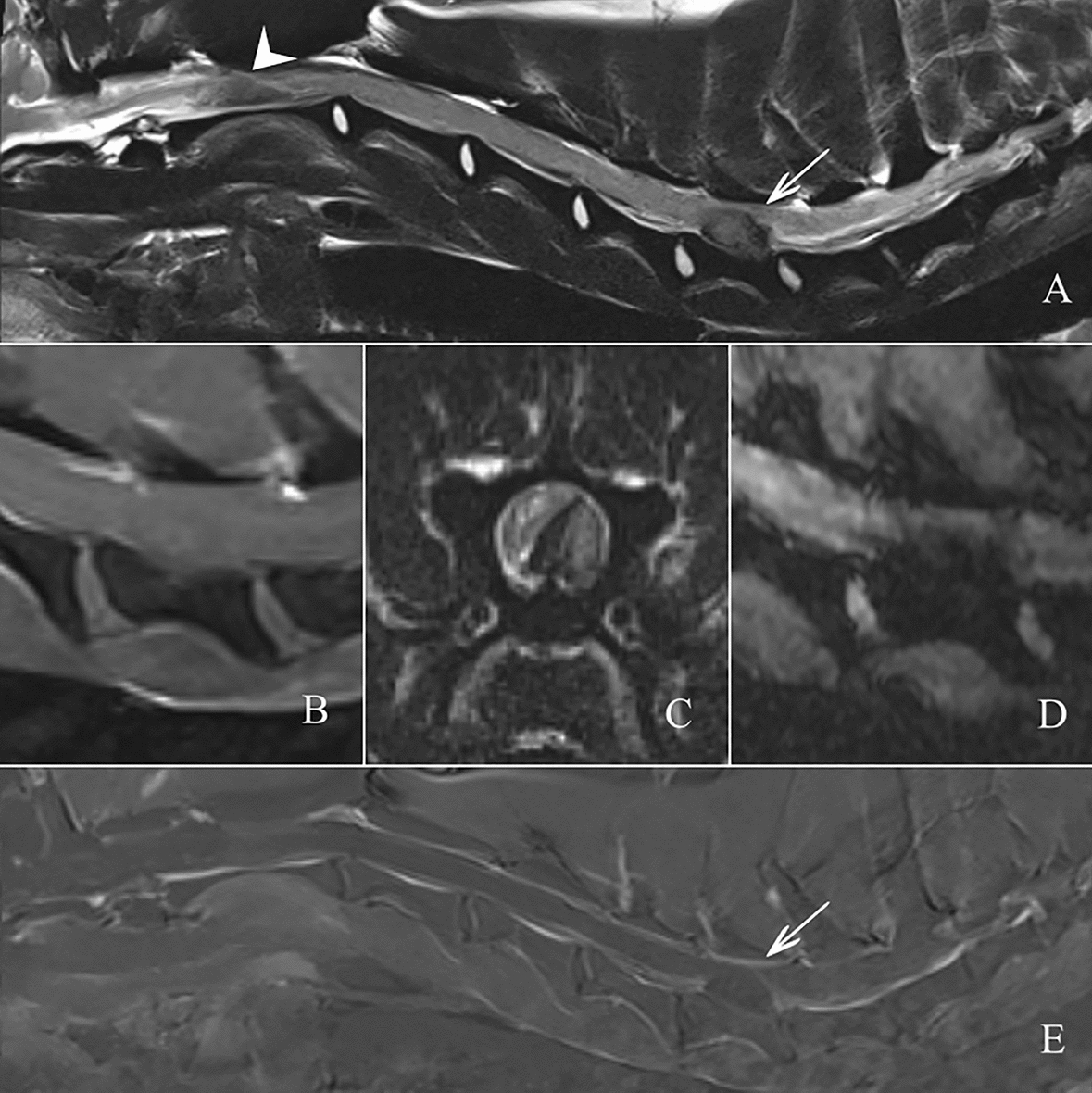
Magnetic resonance imaging of the cervical spine. Extramedullary, extradural, well defined, mildly heterogeneous, oval mass lesion at the level of C6 (arrows). The lesion is hypointense in T2 sagittal (**A**) and transverse (**C**), T1 sagittal (**B**) and mildly hyperintense in STIR. This mass is ventral and right sided, occupying ca. 70% of the vertebral canal with moderate to severe mass effect on the spinal cord that appears displaced towards the left lateral side. The mass shows a mild heterogeneous susceptibility artefact in the sagittal T2 FFE sequence (**D**) and minimal contrast enhancement in the T1 subtraction sequence (**E**). At the level of C2 the spinal cord appears increased in volume. In the CISS sequence there is a circumferential heterogeneous subarachnoideal/subdural material encircling the spinal cord with mixed hyper-hypointense areas. This material is moderate hyperintense in T2 (arrowhead, **A**), STIR and iso- to hypointense in T1. Very mild contrast enhancement is visible (**E**). Susceptibility artifact is noted

Magnetic resonance imaging (MRI) of the cervical spine was performed under general anaesthesia using a 3.0-T scanner (MAGNETOM Verio, Siemens Healthcare) and a commercially available body coil. Multi-planar T2-weighted (T2W), T1-weighted pre- and post-contrast (T1W), fluid attenuated inversion recovery (FLAIR), Gradient Echo, Short Inversion Time Inversion Recovery (STIR) and 3D Constructive Interference in Steady State (CISS) sequences were acquired. The MRI study showed an ovoid extramedullary and extradural, right sided, well demarcated mass lesion at the level of C6 causing a severe compression of the spinal cord, which was displaced to the left. The lesion had a heterogeneous hypointense signal on T2W and T1W images, slightly hyperintense on STIR images and showed a mild contrast enhancement. Moreover, the lesion showed a mild, heterogeneous susceptibility artefact. A similar lesion without compression of the spinal cord was observed at the level of C2 on the CISS images. At this level, a heterogeneous subarachnoidal/subdural material encircled the spinal cord. This material was moderately hyperintense in T2, STIR and iso- to hypointense in T1. Very mild contrast enhancement was visible and a susceptibility artifact was noted as well. Based on the MRI findings, the presumptive diagnosis of subacute, multifocal, epidural haemorrhages was made (Fig. 1). The differential diagnosis included primary and secondary coagulation disorders, infectious and inflammatory disease. CSF was taken from the *cisterna magna* directly after the MRI examination. It was xanthochromic with a severe pyogranulomatous pleocytosis, elevated erythrocyte count (119.196 cells/µL; reference: < 1500 cells/µL) and protein concentration (2470.7 mg/L; reference: < 300  g/L). A leucocyte count was not possible due to massive erythrocyte contamination within the sample. Additionally, the D-dimer concentration was determined by immunoturbidimetry in the CSF and serum samples and was elevated in both (3.68 µg/mL and 3.43 µg/mL, respectively; serum reference: < 0.67 µg/mL). CSF D-dimer from two control dogs presented on the same day were also measured (0.16 µg/mL and 0.09 µg/mL). Given the evidence of epidural haemorrhage, coagulation markers (prothrombin time, partial thromboplastin time, mucosal bleeding time and thrombelastogram) were tested and revealed no abnormalities. The test for *Angiostrongylus vasorum* antigens (Angio Detect) was negative. Based on these findings and in the absence of primary coagulopathies, the presumptive diagnosis of SRMA with secondary, multifocal, epidural haemorrhage was made. In order to confirm the diagnosis, the IgA concentration from serum (137.6 µg/mL) and CSF (5.7 µg/mL) was tested; both were elevated (reference ranges: serum 10.9–100.1 µg/mL; CSF 0.0–0.2 µg/mL). The IgA concentrations were measured by using an enzyme-linked immunosorbent assay (ELISA).

**Fig. 2:**
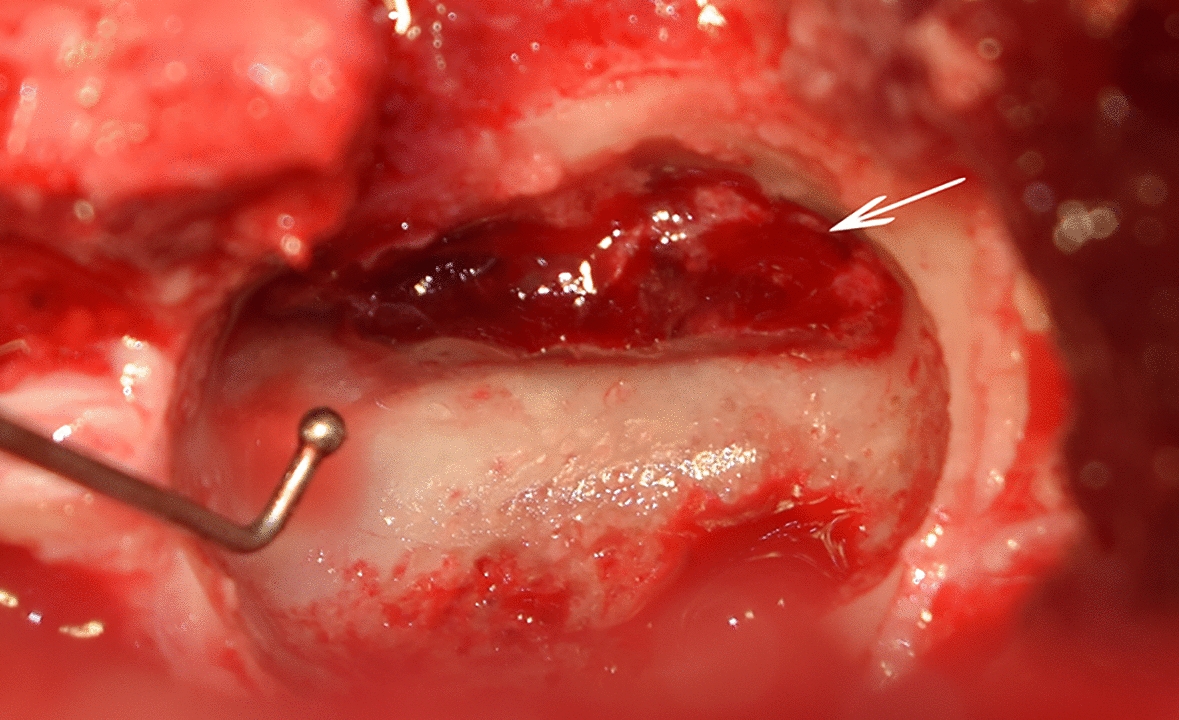
Surgery. Intraoperative image of the surgical field after right sided, partial dorsal laminectomy over C6. The arrow shows the epidural haemorrhage before removal

Due to the severe spinal cord compression, decompressive surgery was performed. The dog received premedication with diazepam (0.5 mg/kg) and atropine (0.015 mg/kg). Propofol (3 mg/kg) was used to induce anaesthesia. General anaesthesia was maintained with isoflurane and fentanyl (40 µg/kg/h). Further medications included ampicillin (50 mg/kg), amoxicillin-clavulanic acid (10 mg/kg) and metamizole (50 mg/kg). A right sided partial dorsal laminectomy at the level of C6 was performed and the epidural haemorrhage was removed (Fig. 2). In order to reduce the likelihood of further bleeding during the procedure, the dog was administered tranexamic acid (10mg/kg, IV).

Post-surgical treatment included immunosuppression with cytosine arabinoside (60 mg/m^2^, SC q12 h over 48 h), fluid therapy using crystalloid solutions (sterofundin 4 mL/kg/h, IV), pain medication (fentanyl CRI at a dosage of 5 µg/kg/h, IV), antibiotics (amoxicillin-clavulanic acid at a dosage of 20 mg/kg, PO q12 h), anti-inflammatory (carprofen at a dosage of 4 mg/kg, PO q24h) and gastroprotective agents (omeprazole at a dosage of 1 mg/kg, PO q24 h).

Treatment with tranexamic acid (12.5 mg/kg, PO q8 h) was continued for 4 days and combined with a constant rate infusion of metoclopramide (15 µg/kg/h), due to its potential emetic effects. Corticosteroid therapy was not started because of its known side effects on wound healing and coagulation. The dog returned to ambulation 4 days after surgical treatment. Cervical pain also significantly improved, and the dog was discharged from the hospital one week after surgery. Prednisolone treatment was started with a dosage of 0.6 mg/kg q24 h after completion of wound healing (12 days after surgery).

At follow-up 4 weeks later, the dog showed a mild proprioceptive ataxia of all limbs. CSF collection under general anaesthesia was repeated. Cytological examination still showed mild blood contamination, with predominance of red blood cells. Prednisolone therapy was continued. Serum C-reactive protein was within the normal limits (1.3 mg/L; reference: < 14.9 mg/L). Eight weeks after surgery, the owners reported a further improvement of the dog’s gait and locomotion. On neurological examination, a mild hypermetric gait of all limbs remained, probably still related to the involvement of the spino-cerebellar tract at the level of C6. The postural reactions were normal, and the dog showed just a mild reluctance to cervical ventroflexion of the neck. Due to the further improvement, prednisolone was further tapered: 0.46 mg/kg q24 h over a further 6 weeks followed by 0.3 mg/kg q24 h over a further 4 weeks. Prednisolone therapy should have been discontinued 6 weeks later but the dog died shortly before for reasons unrelated to SRMA.

## Discussion and conclusions

This is the first case reporting successful combination of surgical and medical treatment in a dog with SRMA and secondary bleeding. Dogs affected with this immune-mediated disorder can develop haemorrhages anywhere in the entire central nervous system; however, these are rarely clinically relevant, as in the case described here [7, 11]. The development of an epidural bleeding was probably due to the associated arteritis, typical of SRMA, and was responsible for a severe compression of the spinal cord at the level of C6. For this reason, the dog needed a surgical decompression. However, since SRMA is primarily a leptomeningitis with the arteritis affecting pia mater and arachnoid mater, an epidural haemorrhage can be considered atypical. Nevertheless, a systemic vasculitis and perivasculitis have been described as well; indeed, this inflammatory process can also involve small to medium-sized arteries in the heart, cranial mediastinum, and cervical spinal meninges (including dura mater) [12], which could account for the presence of an epidural haemorrhage in the dog of our case report.

As aforementioned, the standard treatment protocol for SRMA consists of a long-term immunosuppressive and/or immunomodulatory treatment with prednisolone or prednisone [7]. Nevertheless, steroids are well known for potentially delaying and inhibiting wound healing by interfering with inflammation, fibroblast proliferation, collagen synthesis and degradation, deposition of connective tissue ground substances, angiogenesis, wound contraction and re-epithelization [13]. Moreover, corticosteroids can also interfere with coagulation producing a hypercoagulable state [14], which was undesirable in the dog of the present report who was already receiving tranexamic acid. In order to prevent post-operative healing complications and any other side effects related to the administration of steroids (i.e., gastrointestinal bleedings, interference with coagulation), an alternative therapeutic strategy was developed: the administration of prednisolone was postponed, and immunotherapy was started with cytosine arabinoside. This chemotherapeutic agent is a pyrimidine antimetabolite, which prevents cell proliferation by inhibiting DNA synthesis. In veterinary neurology, it is usually employed in the treatment of meningoencephalitis of unknown origin [15]. With the protocol used here, side effects are very infrequent, nevertheless myelosuppression and gastrointestinal toxicity can occur [16, 17]. Indeed, even if thrombocytopenia has been described in dogs and humans as a consequence of myelosuppression, this occurs only at higher doses than the one used in this report or if cytarabine is used as part of a combination chemotherapy protocol [10, 18–20]. In contrast to corticosteroids, a further influence of cytosine arabinoside on coagulation has not been described so far. This was also confirmed by some human and in vitro studies [21–23]. Moreover, a negative effect on wound healing or rather an antiproliferative effect due to the administration of cytarabine was only demonstrated on ocular tissues in vitro and in vivo (rabbits) [24, 25]. For all these reasons, its use in our patient was considered beneficial and it indeed proved to be an effective therapeutic alternative to corticosteroids in the acute phase of SRMA, which correlates with a previous study [10]. The dog showed a fast improvement of clinical signs and no post-operative complications were observed. During a 4.5 months follow-up-period after surgery, the dog did not show any recurrence of the clinical signs and the owners reported a further improvement of his neurological status at home. Unfortunately, it died shortly before interruption of the prednisolone therapy for unrelated reasons.

An intrathecal fibrinolytic pathway was activated in the CSF of the present dog with SRMA, in agreement with SRMA-dogs in the report by de la Fuente et al. [26]. In these animals, the abnormal high levels of D-dimer can be explained by the presence of vasculitis and sometimes also of subarachnoid bleeding and consequent clot formation [26]. The D-dimer concentration was severely increased not only in the serum but also in the CSF of the dog, considering the ranges obtained for CSF D-dimer in one study that evaluated their concentrations in dogs with various diseases of the central nervous system [26].

Peri- and post-operatively, tranexamic acid, an antifibrinolytic agent [27, 28], was administered in order to reduce the risk of further bleeding. In human medicine, this haemostatic agent, which prevents hyperfibrinolysis, has already been demonstrated as an effective and safe treatment option if used topically or intravenously during spinal surgeries, predominantly in cases with significant anticipated surgical site blood loss [29]. For this reason, its use should also be considered in animals with a high risk of bleeding, e.g. if they undergo spinal surgery.

Coagulation and inflammation are interrelated processes: inflammation can activate coagulation and vice versa, coagulation can affect the inflammatory process [30–35]. Fibrinolysis is the enzymatic process responsible for the removal of intravascular fibrin clots. This process avoids unnecessary intravascular accumulation of fibrin and is an important component of normal haemostasis. The products of fibrin degradation are the D-dimers, which are used as important predictive and prognostic factors in many vascular diseases, like disseminated intravascular coagulation, pulmonary embolism and thrombosis [36]. Indeed, this biomarker could also be employed as a further diagnostic tool when SRMA is suspected, especially while awaiting IgA quantification results in those cases in which the presence of blood contamination in the CSF makes the diagnosis challenging. In fact, haemorrhage could hinder the detection of the typical polymorphonuclear pleocytosis. The parameter could be also used for monitoring the disease. A similar importance can be attributed to the evaluation of CRP, which is normally elevated in the serum of dogs with SRMA in the acute phase of the disease and could also be assessed in the CSF. Indeed, whereas IgA measuring takes many days, CRP and D-dimer quantification takes no longer than a few hours, since it is often available as an in-house test. Further studies are nevertheless required to establish D-dimer concentration ranges in the CSF of normal dogs and to evaluate its concentrations in dogs with acute SRMA in comparison to other central neurological disorders (especially neoplastic and other inflammatory conditions). In this regard, a study already showed that dogs with central nervous system neoplasia or other inflammatory diseases also have increased D-dimer concentrations but still not as high as in animals affected by SRMA [26]. Since reference ranges for CSF D-dimer has not been defined yet, we compared the values obtained in the dog of this report to the ones of the above-mentioned study [26] and considered ours to be increased, even in comparison to the ones measured from the other two dogs presented on the same day in the hospital. These were a 6-year-old female, Cavalier King Charles Spaniel with focal epileptic seizures 4 years after cystoperitoneal shunt implantation due to a supracollicular fluid accumulation (0.16µg/mL) and one 13-year-old neutered female, German Shepard dog with suspected degenerative myelopathy (0.09 µg/mL). However, the significance of this biomarker in dogs with SRMA and secondary haemorrhage in comparison to those affected animals who do not show any evidence of bleeding still has to be evaluated.

In conclusion, in this report a rare case of clinically relevant epidural bleeding secondary to SRMA in a dog was described. In order to support SRMA diagnosis while awaiting IgA confirmation and in presence of blood contamination in the CSF, the evaluation of the D-dimer concentration was used as further diagnostic confirmation. Moreover, the therapeutic protocol used in this case report, which combined surgical decompression and immunosuppressive therapy with cytosine arabinoside, was successful and the dog did not develop any complications. Indeed, cytosine arabinoside administration after surgery proved to be an effective alternative to steroidal immunosuppressive and immunomodulatory drugs in specific cases in the acute phase of the disease.

## Data Availability

The datasets used and/or analysed during the current study are available from the corresponding author on reasonable request.

## Abbreviations

CISS: 3D Constructive Interference in Steady State
CSF: Cerebrospinal fluid
CRP: C-reactive protein
ELISA: Enzyme-linked immunosorbent assay
FLAIR: Fluid attenuated inversion recovery
MRI: Magnetic resonance imaging
SRMA: Steroid responsive meningitis-arteritis
STIR: Short Inversion Time Inversion Recovery
T1W: T1-weighted
T2W: T2-weighted
